# Morphology and Phase Compositions of FePt and CoPt Nanoparticles Enriched with Noble Metal

**DOI:** 10.3390/ma16237312

**Published:** 2023-11-24

**Authors:** Yuri A. Zakharov, Anna N. Popova, Valery M. Pugachev, Nikita S. Zakharov, Irina N. Tikhonova, Dmitry M. Russakov, Vadim G. Dodonov, Denis G. Yakubik, Natalia V. Ivanova, Lilia R. Sadykova

**Affiliations:** 1 Federal Research Center of Coal and Coal Chemistry SB RAS, 650000 Kemerovo, Russia; zakharovya@iccms.sbras.ru (Y.A.Z.); vm1707@mail.ru (V.M.P.);; 2 Institute of Fundamental Science, Kemerovo State University, 650000 Kemerovo, Russia

**Keywords:** nanoalloys, nanocrystals, FePt, CoPt, morphology, phase composition, structural blockiness

## Abstract

The article reveals for the first time the features of nanoparticle morphology, phase compositions, and their changes when heating FePt and CoPt nanoalloys. Nanoparticles were obtained by co-reduction of precursor solution mixtures with hydrazine hydrate. The features were found by a complex of methods of X-ray diffraction (in situ XRD and X-ray scattering), TEM HR, and cyclic voltammetry. In addition, adsorbometry results were obtained, and the stability of different nanocluster structures was calculated by the molecular dynamics method. There were only FCC solid solutions in the X-ray patterns of the FePt and CoPt nanoalloys. According to XRD, in the case of nanoparticle synthesis with Fe and Co content less than 10 at. %, the composition of solid solutions was close to or practically equal to the composition of the as-synthesized nanoparticles quantified by inductively coupled plasma optical emission spectrometry. For systems synthesis with Fe and Co content greater than the above, the solubility limits (SLs) of Fe and Co in Pt were set 11.4 ± 0.7 at. % and 17.5 ± 0.6 at. %, respectively. Therefore, there were non-registered XRD extra-phases (XRNDPh-1) in the systems when C_Fe,Co_ ≥ SL. This statement was supported by the results of TEM HR and X-ray scattering: the smallest nanocrystals (1–2 nm) and amorphous particles were found, which qualitatively agreed with the sorbometry and SAXS results. Molecular dynamics calculations of stability for FePt and CoPt alloys claimed the structures of the most stable phase corresponded to phase diagrams (A1 and L12). Specific peculiarities of the morphology and compositions of the solid solutions of nanoalloys were established: structural blockiness (domain) and composition heterogeneity, namely, platinum enrichment of internal (deep) layers and homogenization of the nanoalloy compositions at relatively low temperatures (130–200 °C). The suggested model of the formation of nanoalloys during the synthesis, qualitatively, was compliant with the results of electrochemical deposition of FePt films on the surface of various electrodes. When nanocrystals of solid solutions (C(Fe, Co) < SL) were heated above specific temperatures, there were phase transformations with the formation of two-phase regions, with solid solutions enriched with platinum or iron (non-registered XRD phase XRNDPh-2). The newly formed phase was most likely intermetallic compounds, FePt_3_, CoPt_3_. As a result of the study, the model was developed, taking into account the nanoscale of the particles: XRDPh (A1, Fe_a_Pt_1−a_) → XRDPh (A1, Fe*_m_*_×a−x_Pt*_m_*_−*m*×a+x_) + XRNDPh-2 (Fe*_n_*_×a+y_Pt*_n_*_−*n*×a−y_) (here, *m* + *n* = 1, *m* ≤ 1, *n* ≤ 1).

## 1. Introduction

An impressive body of research over the last few decades has revealed significant inconsistency between experimentally determined phase compositions (PhCs) of nanosized (NS) bimetallic alloys obtained by chemical co-reduction, and the PhCs pre-defined the phase diagrams (PhDs) of the corresponding systems [1,2,3,4,5,6,7,8]. The most significant difference is the difference in redox potential (Eh) values. This is not irrelevant; as our data indicate, the consideration of this issue for FePt and CoPt nanoalloys, composed of components with a high difference in Eh values, is of considerable interest for elucidating the physicochemical causes of the structural and phase features of bimetallic nanoalloys, in general. The PhDs of bulk systems are complicated and sophisticated, due to existing disordered solid solutions (SSs) of different compositions, intermetallic compounds (IMCs) with different structures, and two-phase regions, which, in turn, lead to an increase in the expected research productivity [7,8].

Along with this, from the practice-oriented point of view, there is the task of obtaining and studying IMCs (FePt, CoPt, FePd, CoPd) with equiatomic compositions having a tetragonal highly ordered L1_0_structure. As is known, IMCs have unique magnetic properties—record coercivity values for bimetallic nanocrystals of 70–90 kOe and high values of saturation magnetization and Curie temperature [9,10,11,12,13]. Therefore, considerable efforts have been directed to obtain such systems (mainly FePt, CoPt) and to study their properties (such as particle morphology and magnetic characteristics), as can be found in the main studies during the last few years [14,15,16,17,18]. Although considerable progress has been made, the current level of achieved coercivity values remains far below predicted characteristics [17,18,19,20,21]. It was also established that FePt nanoalloys might show entirely different characteristics, even though they have the same composition [22]. Such differences can be explained by the variation in atomic ordering and spatial element distribution. The chemical stability and electrocatalytic activity of FePt nanoparticles for the atomically disordered phase differ dramatically from those for the ordered phase [23].

At the same time, a substantial amount of research and development deals with the study of the influence of synthesis methods of desirable nano-IMCs and their thermal treatment modes on the form–size characteristics of nanoparticles (NPs) and their magnetic properties. Neither the peculiarities of phase compositions of synthesized systems nor structural-phase transformations (SPhTs) when NP heated have been studied, particularly with regard to the schemes and models of these processes. Nevertheless, some authors acknowledged the crucial role of the SPhT during phase formation [24,25]. Authors previously reached peculiar conclusions, based on X-ray diffraction studies, about the co-existence of a platinum-rich solid solution as the only observed phase (the X-ray detectable phase, XRDPh) and, along with it, the diffraction-unregistered phase (the X-ray non-detectable phase, XRNDPh) [1,2,26,27].

The above underlines the necessity of an in-depth study of phase states and their features and transformations in FePt and CoPt NPs. Understanding the formation of the ordered L1_0_ structure provides the main framework for establishing the relationship, as well as deep insights into the relationship between the structure and the magnetic properties of FePt and CoPt nanoparticles. The important role of XRNDPh in the formation of the ordered L1_0_ structure was previously emphasized in [3]. So, at the first stage, due to the relative simplicity of the PhCs of FePt and CoPt, it is advisable to consider platinum-rich compositions, which is the focus of the present work. The results of a study of the specificity of FePt and CoPt phase compositions in a wide range of component ratios, as well as the description of SPhTs, will be presented in subsequent works. These further articles will be devoted to the targeted formation of highly ordered L1_0_-type phases. Also, the study of the magnetic properties of FePt and CoPt NPs, revealing hidden relationships between the structure, composition, and magnetic properties of nanoparticles, will be discussed in further works.

## 2. Materials and Methods

### 2.1. FePt and CoPt Nanoparticles Fabrication

The synthesis of FePt and CoPt nanoparticles was carried out in a thermostated reactor at 90 °C by co-reduction of metals by hydrazine from mixtures of freshly prepared aqueous solutions (100 mM) of HPtCl_6_·6H_2_O, FeSO_4_·7H_2_O, CoSO_4_·7H_2_O. Solutions of hydrazine hydrate and NaOH were slowly mixed with precursors’ solutions after their deaeration by argon bubbling at a high rate (2 mL/s). The reactants were constantly stirred (pH = 13–14, process duration 10–15 min). The above synthesis conditions were determined as optimal. In case of deviation from optimal conditions (temperature, hydrazine excess, process rate, pH), there were discrepancies between the desired compositions of NPs and analytically determined compositions; the partial reduction of Fe^2+^ and Co^2+^ caused a relative underestimation of Fe and Co. Similarly, but at 10 ± 2 °C, FePt specimens were synthesized using sodium tetrahydroborate as the reducing agent, without and with the use of ultrasonic stirring during synthesis (500 W, 48 kHz) to blend thoroughly. The duration of the reduction process was at least 5 min. The obtained NPs were washed repeatedly with distilled water, dried at room temperature, and stored in hermetically sealed containers.

### 2.2. Specimen Characterization

The crystal structure, phase compositions, and compositions of NPs were investigated by X-ray diffractometry (XRD). A Bruker D8 ADVANCE A25 powder diffractometer (Bruker, Karlsruhe, Germany) XRD instrument with Cu (Kα) radiation using 40 KV and 40 mA, a Ni filter on a diffracted beam, a size of 0.01° (2θ), and a scan rate of 1°/min were employed. Nanoparticles were placed in a holder and scanned from (2θ) 10° to 90°. This scan range covered all major species of iron, platinum, and their oxides. The high-temperature phase transformations of FePt and CoPt nanoparticles were investigated by “in situ” X-ray diffraction analysis. The experiments were carried out in a high-temperature oven chamber, Anton Paar HTK 1200N (Anton Paar GmbH, Graz, Austria), mounted on powder diffractometer D8 ADVANCE A25. The chamber was evacuated by a turbo-molecular pump, creating a pressure about 10^−7^ mbar. The experimental NPs were uniformly heated without thermal gradients up to 250 °C, 390 °C, 500 °C, and 600 °C (temperature stability ± 0.1 °C) and then exposed for the necessary exposure time. Diffrac.Suite.Eva (V3.1) software was used to analyze the phase composition using the databases ICDD-PDF2 and Crystallography Open Database (COD) with regard to the phase compositions in FePt and CoPt alloys and their crystal lattice parameters (CLPs). The compositions of the detected phases were estimated using empirical relationships between composition (platinum content) and the average volume per 1 atom in the unit cell (V_at_, Å^3^) [3], which was expressed as follows:

(1) $$X_{\mathrm{Pt}}=0.03399V_{\mathrm{at}}^{2}-0.6068V_{\mathrm{at}}+2.411$$

(2) $$X_{\mathrm{Pt}}=0.01260V_{\mathrm{at}}^{2}-0.07845V_{\mathrm{at}}-0.6874$$

In order to determine the SS compositions with high accuracy, a method of locating the diffraction peak in the whole reflection region for CLP measurement, applying the Pearson VII distribution functions to approximate the combined Kα doublet, was used. The position, intensity, and breadth of the Kα1 peak were determined from the intensity measured for the points spanning the Kα doublet. The positions of gravity center of diffraction lines were used to calculate the CLPs.

The Scherrer formula was used to estimate the crystallite size from the broadening of the XRD peaks, following the procedure described in [28].

Small-angle X-ray scattering (SAXS) analysis was conducted with a KRM-1 diffractometer (CJSC Nauchpribor, Novosibirsk, Russia) “at lumen” (Fe-radiation) by counting pulses at points in the range from 0.002–0.348 Å^−1^. SAXS data were used to calculate mass functions of particle-size distribution (Dm(d)). The Tikhonov regularization method was applied for that purpose in the approximation of homogeneous spheres. The particle-size fractions were selected with regard to the approximation of their log-normal distribution [29,30]. Additionally, the estimation of nanoparticle sizes of the most finely dispersed fraction was performed in the Guinier approximation “by the tangent method” [31]. The authors developed a set of software to process SAXS data through accounting for instrumental peak distortions and calculation of size distribution functions [32].

Chemical composition was examined by inductively coupled plasma optical emission spectrometry (ICP-OES), using an iCAP 6500 DUO spectrometer (Thermo Scientific, Waltham, MA, USA). To obtain the best possible sensitivity, the plasma was observed axially, a power of 1150 W. Background corrected signals were used for signal quantification. The contents of the main components and impurities were determined at characteristic wavelengths in the ranges free from spectral and other interferences. The values of element concentrations determined at various analytical wavelengths were averaged. Except for the highlighted cases, the work used mole fractions (mol. %) to define the compositions of specimens.

The ICP-OES results were compared with Fe, Co/Pt ratios determined by energy-dispersive X-ray (EDX) spectroscopy, using JEOL Model JED—2300 attached to a scanning electron microscope with a magnification of 10,000 and with a voltage of 20 KV. 

The morphology of the nanoparticles was studied by scanning electron microscopy, SEM (JEOL Model JSM—6390LV).

The transmission electron microscopy (TEM) investigations were performed on a TEM JEOL 2100 (JEOL Ltd., Akishima, Japan) instrument at an accelerating voltage of 200 KV, the bright-field mode. Specimen suspensions (50–100 µg) were placed on a preparative grid, with a thin layer of carbon applied to its surface. The details of specimen preparation of highly magnetic FePt and CoPt NPs were described in [26]. The measurements of lattice-fringe spacing recorded in high-resolution transmission electron microscopy (HRTEM) micrographs were made using digital image analysis of reciprocal space parameters. The analysis was carried out by the Digital Micrograph software (Gatan Digital Micrograph ver. 3 GMS-III). 

Electrochemical experiments were performed using a VersaSTAT 3 potentiostat/galvanostat with a three-electrode cell (Princeton Applied Research, Oak Ridge, TN, USA). The experimental details were identical to those described in [33].

The specific surface area (SSA) was determined from the nitrogen adsorption–desorption measurements, carried out at 77 K using an ASAP 2020 volumetric adsorption analyzer (Micromeritics, Norcross, GA, USA) after preliminary desorption of trapped gases from the specimens at 105 °C (in accordance with the results of thermodesorption [1]). The BET specific surface areas of the investigated specimens were measured using the standard Brunauer–Emmett–Teller (BET) method. The total pore volumes were calculated using single-point adsorption at a relative pressure of 0.98.

### 2.3. Theoretical Modeling

The calculation of the total energy of clusters was performed by molecular-dynamics simulations using the LAMMPS program [34] and a modified embedded-atom method (MEAM) (consider the second nearest-neighbor interactions, interaction potential MEAM-2NN) [35]. The potential parameters were taken from the repository of interatomic potentials [36]. The initial shapes of the nanoclusters were set as regular polyhedra, consistent with the PhDs of the systems [8,24], structures established from XRD-data (FCC A1-type disordered phases and L1_2_-type ordered phases) (see below), and interatomic distances characteristic of bulk specimens. The simulations were performed within the NVT ensemble framework using the Nose–Hoover thermostat; the thermostat constant was 1.5 × 10^−15^ s.

The first stage of the simulation involved relaxation of the nanocluster structure at a temperature of 0.001 K, with a dwell time of 5.0 × 10^−12^ s. The next step was to heat to 1000 K at a rate of 40 K/ps.

## 3. Results

Table 1 shows the compositions of the synthesized specimens as determined by chemical analysis and, for comparison, calculated from the lattice parameter by X-ray diffraction.

According to chemical analysis, the determined compositions for the platinum-rich specimens were almost identical to the assumed composition. Ni, Cu, Zn, and Si were found as impurities, the total content of which did not exceed 0.2 wt%. Further compositions are provided (except for the noted cases) according to the results of ICP-OES.

### 3.1. X-ray Detectable Phase—FCC (A1-Type Phase) Solid Solution, XRDPh

**Figure 1:**
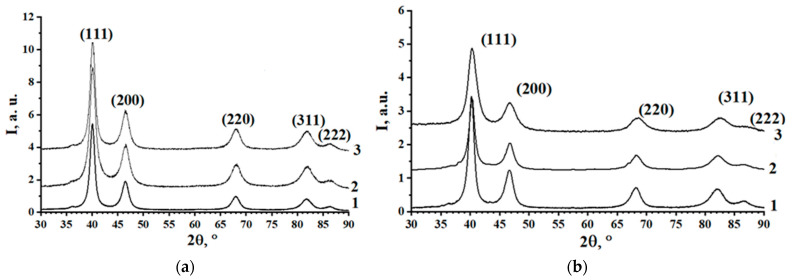
XRD patterns of FePt (1—Fe_7_Pt_93_; 2—Fe_13_Pt_87_; 3—Fe_15_Pt_85_) (**a**) and CoPt (1—Co_14_Pt_86_; 2—Co_21_Pt_79_; 3—Co_20_Pt_80_) (**b**) specimens.

**Figure 2:**
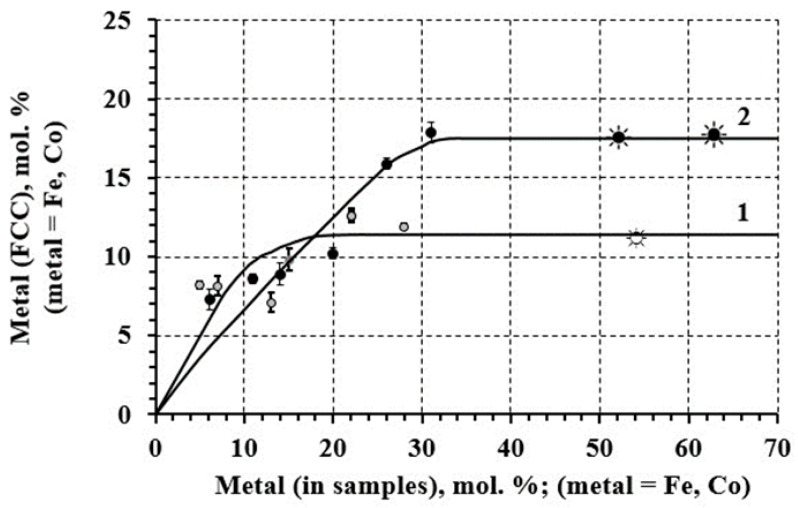
Dependence between FCC solid-solution composition (according to XRD) and composition of the specimens (according to ICP-OES): FePt (1) and CoPt (2). (☀, ☼—not discussed in the paper; shown for information, discussed in further papers; Vertical line crossed the bullets shows error-bar values).

**Figure 3:**
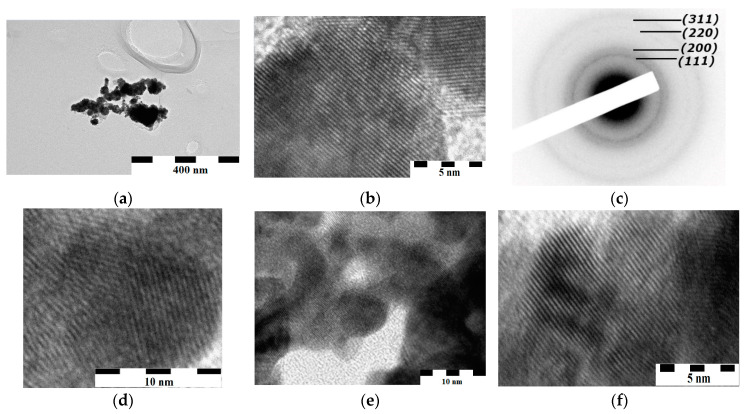

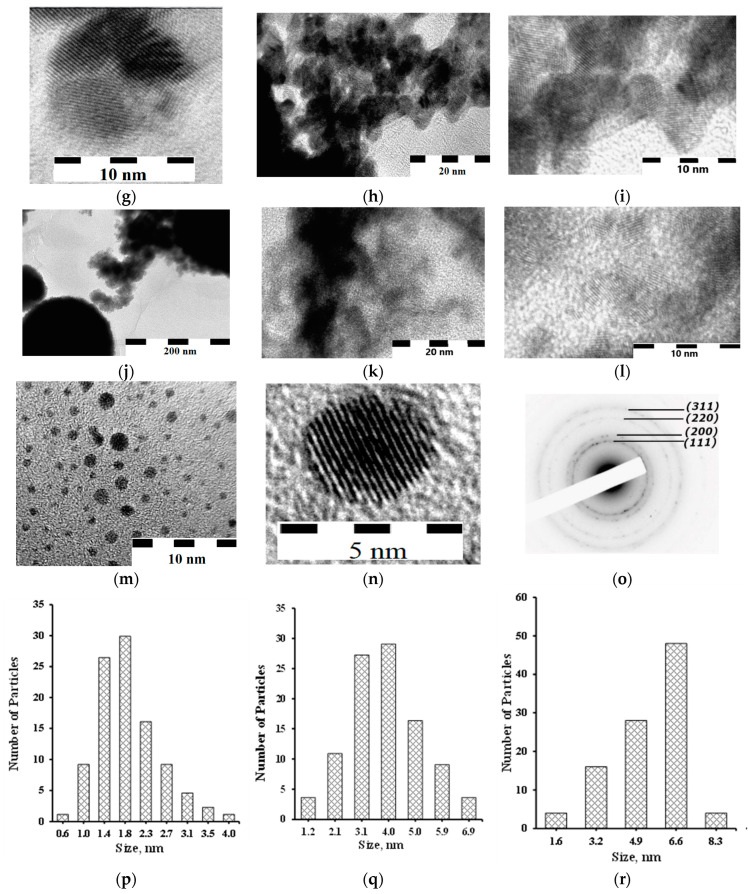
HRTEM images (Co_26_Pt_74_ (**a**,**b**), Co_31_Pt_69_ (**d**–**f**), Fe_13_Pt_87_ (**g**–**i**), Fe_28_Pt_72_ (**j**–**l**), Fe_12_Pt_84_ (synthesis with a sodium tetrahydroborate as a reducing agent) (**m**,**n**))*,* the size distribution (Fe_12_Pt_84_ (synthesis with a sodium tetrahydroborate as a reducing agent)) (**p**), Fe_13_Pt_87_ (**q**), Co_31_Pt_69_ (**r**), and SAED images (Co_14_Pt_86_ (**c**) and Fe_15_Pt_85_ (**o**)) of nanoparticles.

According to XRD patterns (Figure 1a,b; also see the Supplementary Materials, Figures S1 and S2), nanoparticles are represented by a single phase, FCC (A1-type phase) solid solution—XRDPh. The calculated CLP values and the compositions of FCC phases calculated using CLP and relations 1 and 2 in comparison with the total compositions of specimens are provided in Table 1. The calculations, for clarity, are presented in the form of the dependencies of iron and cobalt content (C(Fe) and C(Co)) in the FCC phase on their total content in the specimens, as shown in Figure 2. According to the results, the XRDPhs are platinum-rich SS, with upper (at synthesis) solubility limits (LSs) of Fe and Co in FCC solid solution, 11.4 ± 0.7% and 17.5 ± 0.6%, respectively. Observed phases are in full compliance with the PhDs of FePt, CoPt systems [8,24]. Moreover, FCC structure of the synthesized NPs is also confirmed by SAED data (see below, Figure 3c,o).

Typical microphotographs of FePt, CoPt nanoparticles, as well as their size distributions, are presented in Figure 3. There are parallel bands in all TEM images, which can be naturally attributed to lattice fringes (Figure 3c–g,i,m). The most represented distances between lattice fringes are 2.4 ± 0.1 Å. The calculation of lattice-fringe distances in the [110] direction for the (111) and (100) planes, and in the [100] direction for the (001) plane of the FCC lattice, yields the following values: for the case of [110]/(100)—2.75 and 2.74 Å (CLP × (1/2)^0.5^); for [110]/(111)—2.39 and 2.37 Å (CLP × (3/8)^0.5^); and for [100]/(001)—1.96 Å (CLP × 0.5) (the first values are for the FePt system; the second values are for the CoPt system; the mean values of the CLPs are 3.901 Å and 3.873 Å for FePt and CoPt, respectively). 

The observed lattice-fringe distances agree with the CLP values calculated from the XRD data in the [110] direction for the (111) plane of the FCC lattice; for other variants, discrepancies are observed. It can be concluded that the (111) crystalline faces are predominantly developed in the obtained SS. 

Compositions of the SSs, determined more precisely by XRD, are consistent with findings from SAED, determined with a minor accuracy due to the blurring of diffraction rings (e.g., for Fe_15_Pt_85_ and Co_6_Pt_94_ 9 ± 0.9% (XRD), 8 ± 2.0% (SAED) and 6 ± 0.5% (XRD), 4 ± 2% (SAED), respectively, Fe and Co).

**Figure 4:**
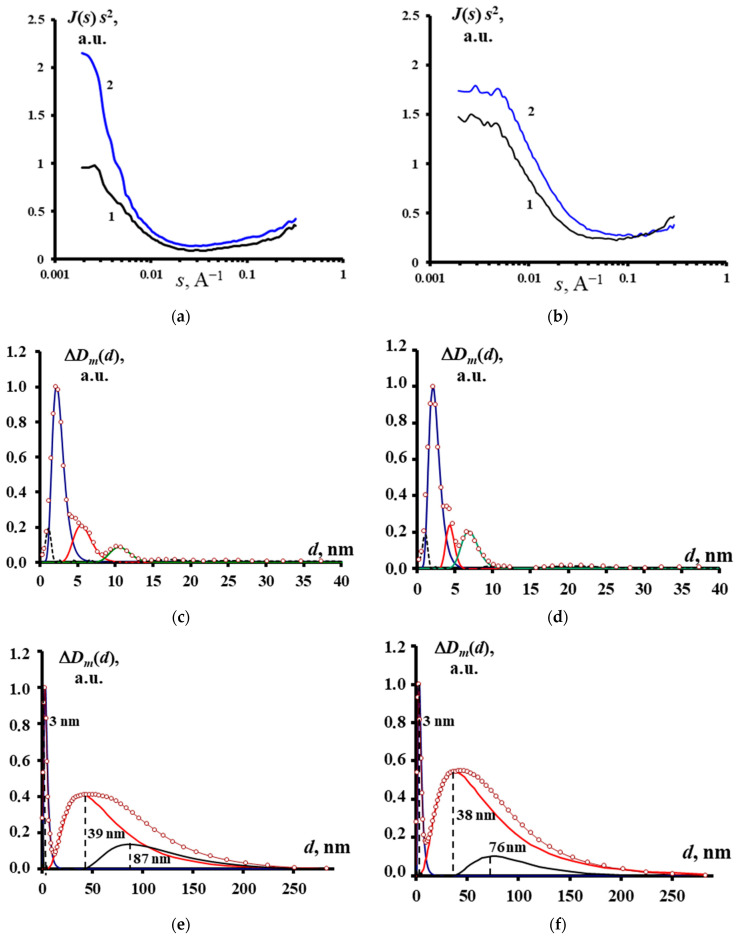
SAXS data of FePt and CoPt specimens ((**a**), 1—Fe_22_Pt_78_, 2—Fe_7_Pt_93_; (**b**), 1—Co_19_Pt_81_, 2—Co_24_Pt_76_); mass functions of particle size distribution (Dm(d)) of FePt and CoPt specimens ((**c**)—Fe_7_Pt_93_, (**d**)—Fe_22_Pt_78_, (**e**)—Co_19_Pt_81_, (**f**)—Co_24_Pt_76_).

The sizes of the FePt and CoPt nanoparticles, synthesized under the similar conditions, were close (maxima of size distributions are in the region of 3–6 nm, Figure 3q,r) and practically corresponded to the fraction of inhomogeneities observed by the SAXS method (Figure 4c,d), as well as to the crystallites sizes calculated using the Scherrer equation (6–10 nm). According to TEM and SAXS data (Figure 3a,b,e,h,j, Figure 4e,f), crystallites formed aggregates with sizes in the region of 30–150 nm and somewhat larger for the CoPt system, which in turn formed submicron-sized agglomerates.

In addition to the described crystallites (the major fraction), their aggregates, and their agglomerates, TEM images showed nanocrystals fractions with sizes of 1–3 nm (for the FePt system—Figure 3l–p), with parallel bands, indicating their crystallinity (Figure 3l,n). Their sizes corresponded to the sizes of the inhomogeneities fractions (2–5 nm) according to SAXS (Figure 4c,d), and lattice-fringe distances (2.4–2.42 Å) (Figure 3l) were practically similar to the values of the major fractions (Figure 3i–k); thus, they were FCC solid solutions with a developed (111) crystalline face.

The analysis of TEM images and XRD data led to the conclusion about the complex structure of nanoparticles.

First, according to the TEM images, a part of NPs is composed of structural blocks (Figure 3d,g); in fact, the blockiness is more developed for CoPt and for FePt obtained by using sodium tetrahydroborate as a reducing agent. The structural blockiness of FePt nanoalloys obtained by another method is a known phenomenon [37,38,39]. As nanoblockiness of magnetic bimetals noticeably affects their magnetic characteristics, including their coercivity [37], it is necessary to control this effect during the formation process of the L1_0_-phase at the heating of the specimen.

In addition to the structural peculiarity of the NP structure that has been mentioned, a cycle of experiments with the analysis of peak profiles, performed to precisely estimate CLP values and the dynamics of their changes, using expressions (1) and (2) (to estimate the changes in the NP compositions when specimens were heated up to ≈ 390 °C), allowed us to reveal the variability of the compositions along the depth of the NPs and the variability of the averaged NP compositions.

**Figure 5:**
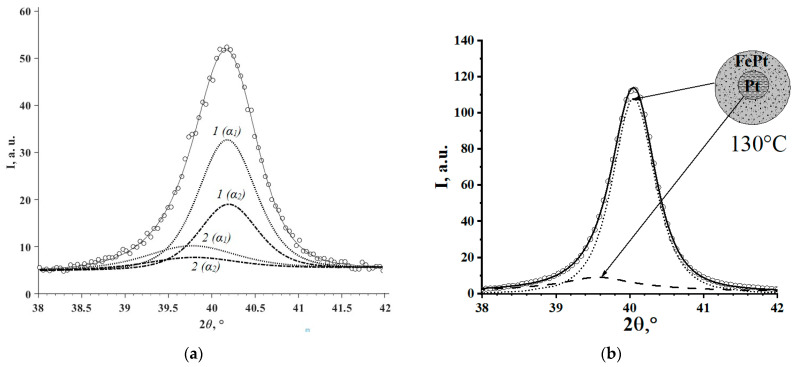

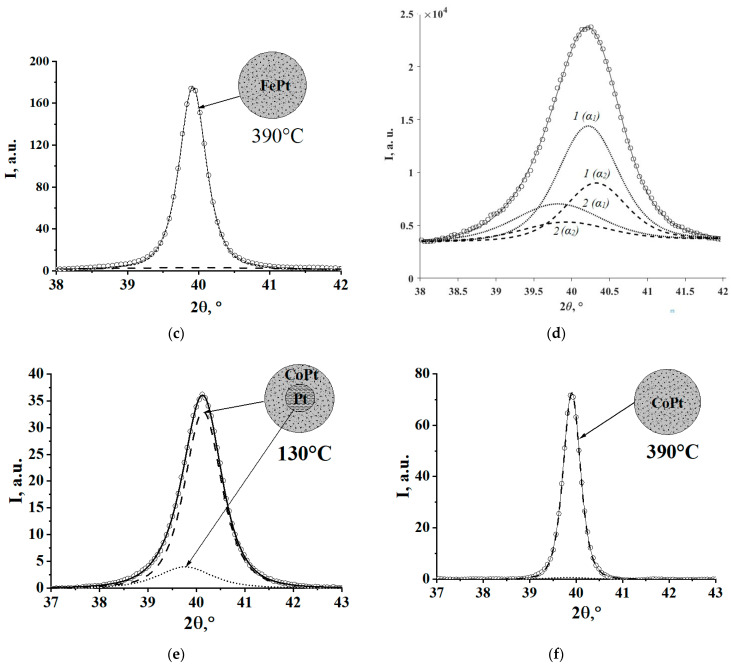
Deconvolution of the (111) peak profiles for specimens Fe_5_Pt_95_ (**a**–**c**) and Co_6_Pt_94_ (**d**–**f**) by the Pearson VII distribution functions (taking into account factors of [28] and approximation of the combined Kα doublet at 30 °C (**a**,**d**), 130 °C (**b**,**e**), and 390 °C (**c**,**f**); ○—experimental data; dotted line—«main» peak; dashed line—Pt-rich phase; solid line—approximation.

Due to the deconvolution of the weakly asymmetric peak profiles (Figure 5), a low intensity peak, shifted relative to the main peak toward smaller angles 2θ, was identified. Its intensity decreased with increasing temperature until it disappeared at T ≥ 390 °C. It showed that the peak of the almost purely “platinum” phase became virtually unobservable at particle sizes smaller than 2 nm.

**Figure 6:**
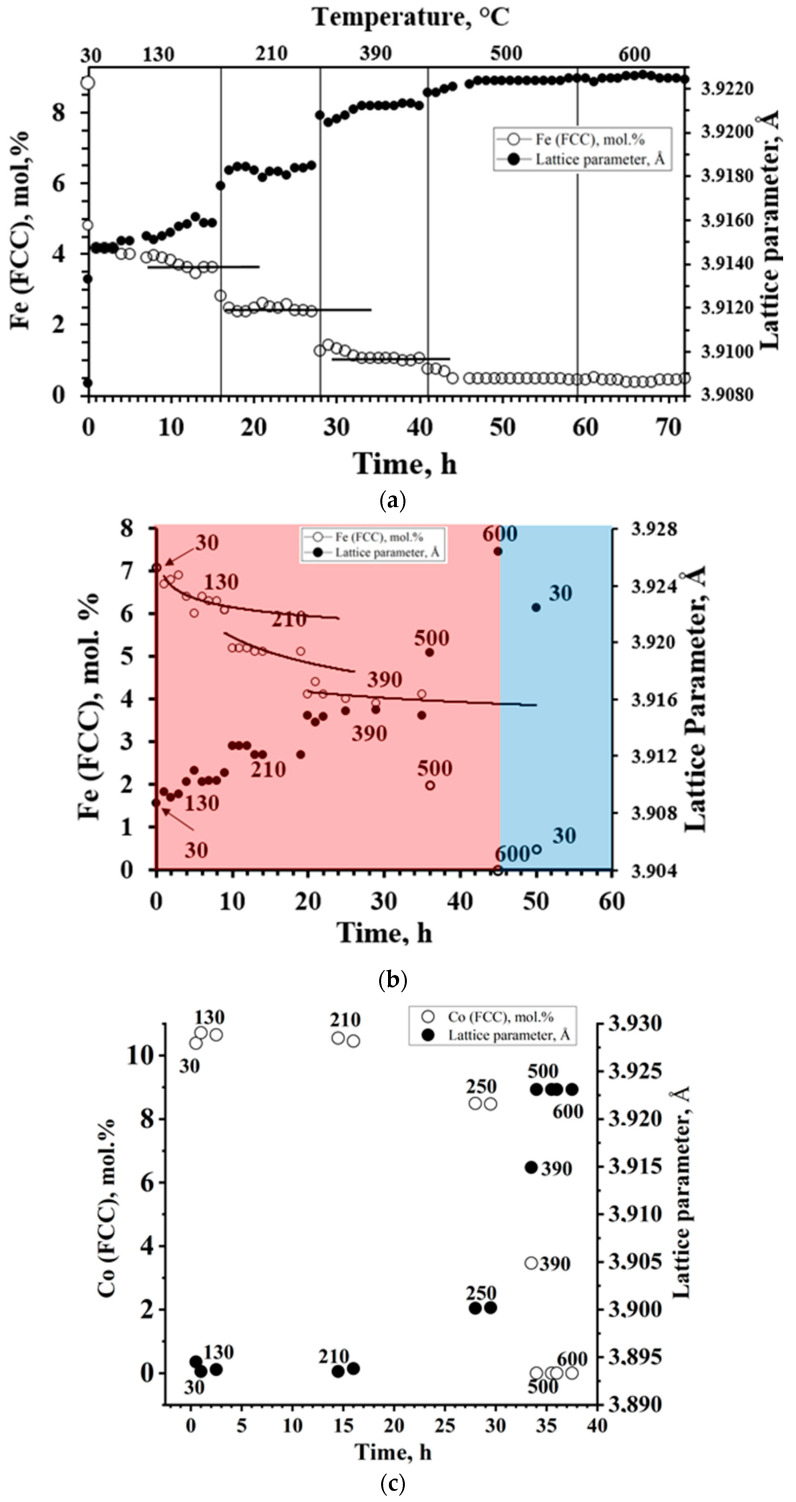
Typical dependence between temperature–time and lattice parameter–content of Fe (**a**,**b**) and Co (**c**) in FCC solid solution (temperatures (°C) are above markers) (Fe_15_Pt_85_ (**a**), Fe_5_Pt_95_ (**b**) (red and blue regions correspond to heating and cooling, respectively), Co_14_Pt_86_ (**c**)) (error-bar values of the lattice parameters are much less than the bullets).

The results presented in Figure 6 indicate a complex process of changes in the CLP of the FCC solid solution when the specimens were heated.

Approximately following the same scenario, changes in CLP occurred when a rapid stepwise increased in temperature, followed by prolonged thermostatization at each step. When T ≤ 250 °C, at first, CLP sharply increased, proceeding within 0.5–1 h, and when 250 < T ≤ 390 °C, there was a slow and less noticeable further increase in the parameter until reaching practically constant values. When T ≥ 390 °C after the induction period, CLP significantly increased, close to the CLP of individual Pt at thermostatization. The CLP values and the Fe and Co contents in the SS were provide to 30 °C. They were calculated based on the values of thermal expansion coefficients of crystal lattice established in special experiments. Therefore, CLP represented only the effects associated with changes (during heating) in NP compositions, i.e., an increase in the Pt content. The nature of the effect at the first stage of heating is discussed below. The increase in Pt content in XRDPh at the second stage was due to the existence in NPs internal (deep) platinum-rich sub-regions and the general homogenization during the heating of NP compositions. These effects were insignificant: Fe content in NPs after heating at 130, 210, and 390 °C decreased (in general) by 1.0–1.2% and 1.1–1.4%, and by ≈ 0.8% for specimens Fe15Pt85, Fe13Pt87, and Co14Pt86, respectively (Figure 6).

The CLP increases, determined by changes in the position of the centers of gravity of the XRD-patterns, were indicative of changes in SS compositions: this suggested there was a source (or sources) of Pt in the form of undetectable (by XRD) phases that were “platinum nuclei” in the depth of NPs or separate platinum-rich NPs. Such a hypothesis is confirmed by the overestimation of Fe and Co content in the synthesized specimens relative to those calculated from XRD data; it is more noticeable in the high Pt content region (Figure 2). The NP compositions averaged at elevated temperatures, due to homogenization. Based on these data (Figure 2), the excess of Fe, Co content in non-homogenized-by-NP composition was 1–2%, which was close to the estimates made above.

A simplified assessment of the main (111) reflection of FCC phase for Fe5Pt95 and Co6Pt94 (Figure 5) showed the co-presence of a minor amount of additional FCC phase in NPs, besides major FCC phase: (1) 25 ± 5% and 15 ± 5%, with Fe and Co content of ≈0.4 and 0.3 mol.%, respectively, and (2) 75 ± 5% and 85 ± 5%, with Fe and Co content of 8 and 7 mol. % (which corresponds to Figure 2), respectively. It followed (taking into account material balance) that the averaged content of Fe, Co in the specimens was 6–7% and 5–6%, respectively, which agreed well with the ICP-OES results of the of the averaged composition analysis of these specimens (Table 1).

Therefore, there are non-trivial effects; that is, the variability of NP compositions, both internal (radial) variability, due to the presence of sub-regions enriched in platinum, and variability of NP compositions. The specificity of bimetal nanoparticle formation by redox processes at essential redox potential differences of precursors was the most likely reason for such effects (E(PtCl_4_^2−^/Pt^0^) = +0.76 V, E(Fe^2+^/Fe^0^) = −0.44 V, E(Co^2+^/Co^0^) = −0.28 V, according to [40]). It consisted in a significant decrease in Eh during the formation of metal nuclei (clusters) from the atoms, depending on a number of factors [41,42]. This effect was well-known and used in scientific photography when discussing the formation of a latent image (with Ag clusters) [43].

In our case, a significantly more active (strong) reduction of Pt led to a decrease in the Eh difference between Pt clusters enlarging at the early stages of synthesis and the Fe, Co (atoms) subsequent co-reduction of components, with the formation of platinum-rich SSs as a result. It is otherwise with independent (separate) reduction of components, which would lead to the sequential formation of practically individual phases of Pt and Fe (Co), due to high differences in the Eh values of metals. The observed asymmetry of the XRD reflections, representing the formation of NPs of different compositions, required the introduction of extra assumptions, which complicated the model. Variability in the composition of NPs could exist due to the formation of NPs as a result of the co-reduction of [PtCl_4_]^2−^ and Fe^2+^ (or Co^2+^), also, on the surface of Pt nuclei. And vice versa, the process could accompany the peeling off from the surface of Pt nucleus nanoparticles of solid solutions. Due to a decreased Eh of [PtCl_4_]^2−^ and Fe^2+^ (or Co^2+^) to values below the Eh of the Pt nucleus, there was reduction in [PtCl_4_]^2−^ and Fe^2+^ (or Co^2+^) on the surfaces of Pt nucleus nanoparticles. As a result, a solid solution with a higher content of Fe or Co was formed.

These hypotheses will be verified in experiments on the transformation of XPS spectra in the process of the Ar-etching of specimens.

A series of experiments on the precipitation of nanothickness FePt films confirmed the above model.

**Figure 7:**
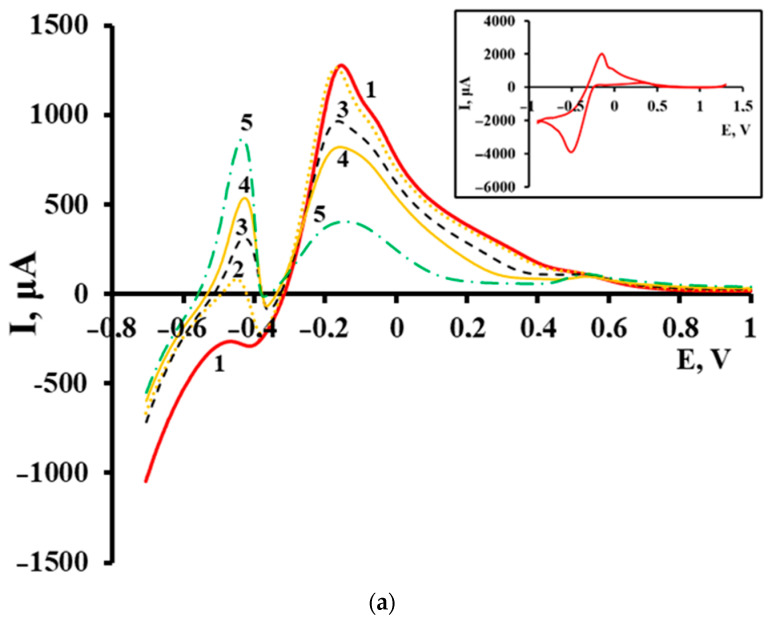

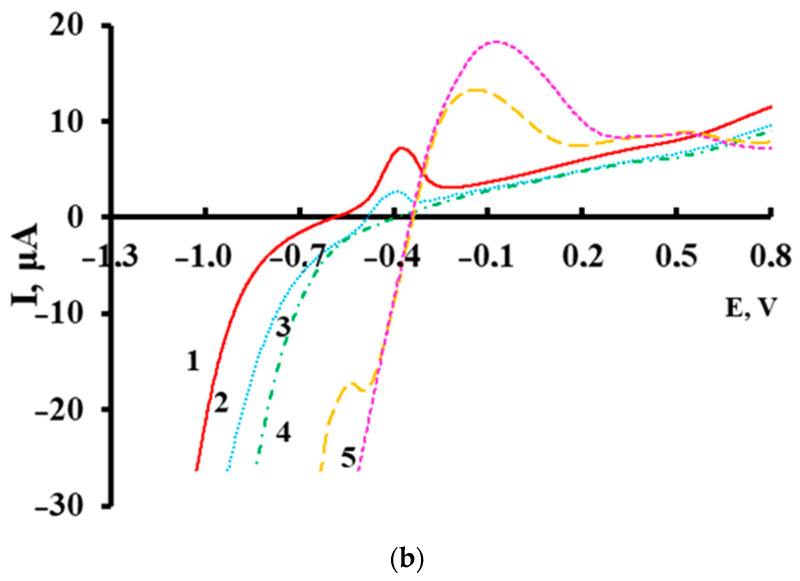
Current-voltage diagrams, measured in acid chloride electrolyte (**a**) with pre-deposited platinum of c(PtCl_6_^2−^) = 6 × 10^−6^ mol/L and subsequently deposited iron of c(Fe^2+^), 10^−4^ mol/L: 1–0.2; 2–0.4; 3–0.8; 4–1.6; 5–2 (inserts in (**a**)—cyclic current-voltage graph of electrolytic deposited platinum from solution c([PtCl_6_]^2−^) = 6.6 × 10^−6^ mol/L); (**b**) deposition of iron from mix of solutions c(Fe^2+^) = 6.6 × 10^−4^ mol/L (1–5) and c([PtCl_6_]^2−^), 10^−7^ mol/L: 2–4.1; 3–8.1; 4–28; 5–68.

The voltammetric curves of anodic oxidation of pre-electrolytically formed FePt films (Figure 7) clearly showed peaks in the regions (−0.5)–(−0.3) V, (−0.2)–(+0.2) V, and (+0.55) V. From the comparison of Figure 6a,b and also according to [44] the broad peak in the region (−0.2)–0 V, with a noticeable right shoulder structure, belonged to electrocatalytic hydrogen oxidation. SSs acted as catalysts, as evidenced by the decrease in catalytic activity with increasing Fe content in the nanoparticles. Matching the characteristics of the peaks in the regions of high potential and in the region (−0.5)–(−0.3) V with XRD data allowed us to attribute them to the oxidation of the particles of platinum-rich SS and to the oxidation of an intermetallide of variable composition (FePt or, more likely, Fe_3_Pt) containing a significant proportion of iron. Arguments in favor of this are the location of peaks on the potential scale and the result of matching peak characteristics with XRDdata:

- there was practical independence between the component ratio and the position and intensity of the peak in the +0.55 V region, which was in agreement with a small solubility limit of Fe in Pt (Figure 2) and with an insignificant variation of the amount of solid solution (intensity of diffraction peaks) when changing the component ratio;
- the analysis’s XRD results were consistent both with a marked increase in the amount of intermetallic and increasing Fe content in it with the increase in its total content in the system (according to the direction of the peak maximum shift in the region (−0.5)–(−0.3) V) [1,27].

A more precise identification of the anodic peaks is the subject of our special studies. 

Within the framework of the issue under discussion, an important conclusion is the following. Electrochemical reduction from a mixture of precursor solutions (similar to those used in the synthesis of FePt nanoparticles) in the presence of Pt NPs preformed on the electrode leads, based on the anodic voltammetry, to the formation of solid-solution as well as intermetallic compounds. This was observed in the case when the Fe^2+^ content in the solution was ≥30%, which was higher than the LS of Fe in Pt. At the same time, during the electroreduction of the same precursors at the minimum possible process time and current on the cathode containing no preformed Pt clusters, almost pure Pt phase was formed, even at molar ratios of Fe^2+^/[PtCl_6_]^2−^ up to ≈300–350.

The results in Table 1 and Figure 2 show that when the Fe and Co contents below the LS the elemental compositions of the SSs were close, or practically equal to the molar ratios of metals in the precursor solutions, the Fe and Co contents in the SSs were practically equal (or close) to their contents in the specimens. The conditions of electrodeposition of mixed (IMCs and SSs) phases of Fe-Pt and chemical reduction of solid solution were not identical (temperature, pH of reaction medium, times of process), but we hold the view this could not be the reason for the noted significant differences. The schemes of electrochemical and chemical reduction are different.

In view of the above, the scheme of nanoparticle formation, according to the obtained results, was as follows (by the example of the FePt alloy):

Formation of Pt clusters (fast stage):

(3) $$I.\;{\left[{\mathrm{PtCl}}_{6}\right]}^{2-}+N_{2}H_{4}+4{\mathrm{OH}}^{-}\to \mathrm{Pt}+4H_{2}O+N_{2}+6{\mathrm{Cl}}^{-},$$

(4) $${\mathrm{Pt}}^{0}+{\left[{\mathrm{PtCl}}_{6}\right]}^{2-}+N_{2}H_{4}+4{\mathrm{OH}}^{-}\to {\mathrm{Pt}}_{2}+4H_{2}O+N_{2}+6{\mathrm{Cl}}^{-},$$

(5) $${\mathrm{Pt}}_{n-1}+{\left[{\mathrm{PtCl}}_{6}\right]}^{2-}+N_{2}H_{4}+4{\mathrm{OH}}^{-}\to {\mathrm{Pt}}_{n}+4H_{2}O+N_{2}+6{\mathrm{Cl}}^{-}.$$

According to the reaction (5) at the final stages of Pt-cluster formation, the reduction of Fe^2+^ and Co^2+^ and their adsorption in insignificant amounts were also possible.

II. Intermediate reactions were probable to proceed as:

(6) $${\left[{\mathrm{PtCl}}_{6}\right]}^{2-}↔\;{\left[{\mathrm{PtCl}}_{4}\right]}^{2-}+2{\mathrm{Cl}}^{-},$$

(7) $${\left[{\mathrm{PtCl}}_{4}\right]}^{2-}↔{\;\mathrm{Pt}}^{2+}+4{\mathrm{Cl}}^{-},$$

as the process was being carried out in an alkaline environment:

(8) $${\left[{\mathrm{PtCl}}_{4}\right]}^{2-}+2{\mathrm{OH}}^{-}\overset{100{}^{\circ}C}{\to }\mathrm{Pt}{\left(\mathrm{OH}\right)}_{2}↓+6\mathrm{NaCl}+2H_{2}O,$$

(9) $${\mathrm{Fe}}^{2+}+2{\mathrm{OH}}^{-}↔\;\mathrm{Fe}{\left(\mathrm{OH}\right)}_{2}.$$

The realization of reactions (6)–(9) depended on the values of the solubility products $\mathrm{Fe}{\left(\mathrm{OH}\right)}_{2}$ and ${\mathrm{Na}}_{2}\left[\mathrm{Pt}{\left(\mathrm{OH}\right)}_{6}\right]$ and the instability constants ${\left[{\mathrm{PtCl}}_{x}\right]}^{2-}$, as well as on their rates and stages of reduction of the components (10) and (11).

III. The adsorption of [PtCl_4_]^2−^ and Fe^2+^ ions on the surface of Pt clusters and their sequential reduction to form a Pt-rich solid solution was as follows:

(10) $${\mathrm{Pt}}_{n}\begin{array}{c} \overset{+{\left[{\mathrm{PtCl}}_{6}\right]}^{2-}}{\to }\\ \underset{+{\mathrm{Fe}}^{2+}}{\to } \end{array}{\;\mathrm{Pt}}_{n}\cdot {\left[{\mathrm{PtCl}}_{6}\right]}_{\mathrm{ads}.}^{2-}\cdot {\mathrm{Fe}}^{2+}{}_{\mathrm{ads}.},$$

(11) $$2N_{2}H_{4}+8{\mathrm{OH}}^{-}+\left\{\begin{array}{c} Pt_{n}\cdot {\left[{\mathrm{PtCl}}_{6}\right]}_{aдc.}^{2-}\\ Pt_{n}\cdot {\mathrm{Fe}}_{aдc.}^{2+} \end{array}\right\}\begin{array}{c} \overset{K_{1}}{\to }\\ \overset{K_{2}}{\to } \end{array}\;\mathrm{Fe}\cdot {\mathrm{Pt}}_{n+1}+2N_{2}+8H_{2}O+6{\mathrm{Cl}}^{-},$$

where K_1_ and K_2_ were effective rate constants of reduction reactions.

The high stability (Kd) of ${\left[{\mathrm{PtCl}}_{4}\right]}^{2-}$ and the low rates of the “religanding” process (8) [45] suggested that the electrochemical reduction of Pt was exactly from the complex ${\left[{\mathrm{PtCl}}_{4}\right]}^{2-}$. It could be assumed that the processes (8) were unlikely. 

This assumption was supported by experimental results that indicated the same compositions of SS to the ratios of the components in precursor solutions (Table 1, Figure 2) when the Fe and Co contents in nanosystems were below the LS. Furthermore, it allowed us to consider processes (8) and (9) as unrealizable in our conditions—due to both the high difference in solubility products of Fe(Co)(OH)_2_ and Pt(OH)_2_ and the low-solubility product values (Fe(OH)_2_—8·10^−16^, Co(OH)_2_—6.3·10^−15^, and Pt(OH)_2_—1·10^−35^) [46]. In addition, these results (Figure 2) also allowed us to consider the schemes of electrochemical and chemical reduction of Fe^2+^, Co^2+^ as non-equivalent and add the stage of their sorption on the formed Pt clusters, which provided for bringing the Eh values of ${\mathrm{Fe}}_{\mathrm{ads}.}^{2+}$ (${Co}_{\mathrm{ads}.}^{2+}$), ${\left[{\mathrm{PtCl}}_{4}\right]}_{\mathrm{ads}.}^{2-}$ closer together, thus providing convergence of their reduction rates.

It follows from the above that with the sequential process (10), the parallel nature of the reduction process (11), and its limiting role, the ratio of K1 to K2 was close to 1 (K1/K2 ≈ 1).

### 3.2. Phase Transformations When NP Specimens Heated

**Figure 8:**
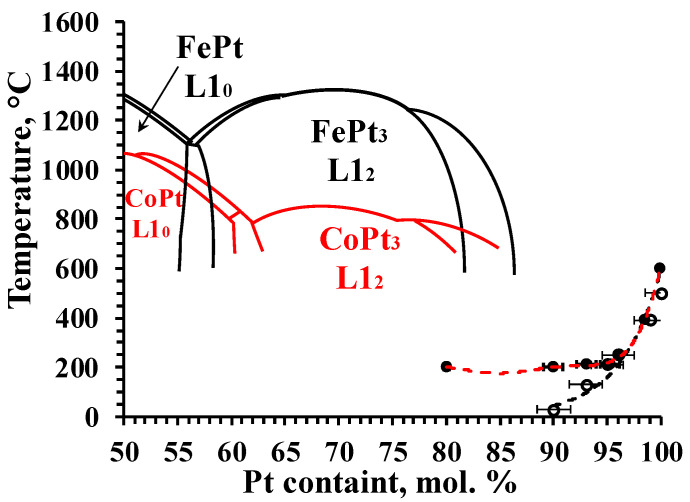
Combination of fragments of phase diagrams of Pt-rich alloys (CoPt—red solid lines and FePt -black solid lines). There are the temperature boundaries of FCC solid solution for FePt (black dashed lines), CoPt (red dashed lines), shown with variation of experimental data, and the hypothetical boundary of the two-phase region.

In [1], it was shown that when heating to ≈600 °C, FePt and CoPt nanoalloys, synthesized by the method described above, formed a two-phase region. Phase composition was the X-ray detectable phase, XRDPh—the platinum-rich FCC solid solution—and the X-ray non-detectable phase, XRNDPh-2—probably (since not confirmed by XRD) the FCC solid solution enriched with Fe (Figure 8). Also, a phase transformation model was proposed, according to which the highly dispersed particles (or clusters) of XRNDPh-2 were formed on (or close to) the surface of XRDPh nanoparticles by the transfer of SS crystallites from the deep regions of nanoparticles to their surface, i.e., at distances ≤ 3–4 nm (Figure 3q,r). Such a process was possible at the specified temperatures, due to the appropriate diffusion and self-diffusion coefficients of Fe, Co, and Pt. The observed effects and the proposed model were unusual in that, first, the phase transformations were observed at such low temperatures and, moreover, with relatively high rates. To explain it based on their nano-sizes, the model has to carry out an extra study to obtain additional supporting data.

**Figure 9:**
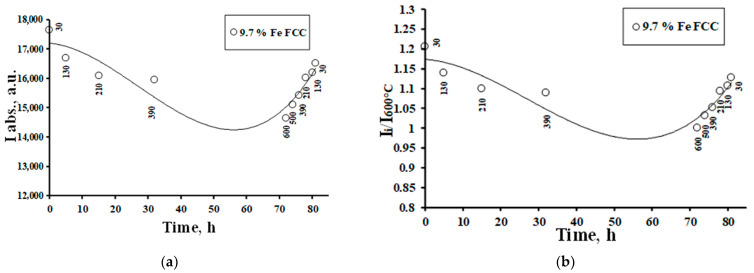

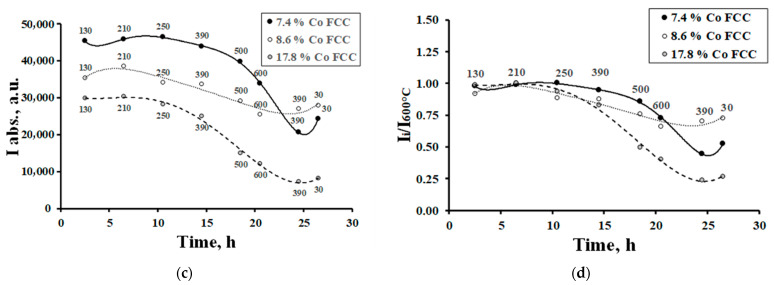
Integral intensities (**a**,**c**) and I_i_/I_600°C_ ratios (**b**,**d**) of the diffraction peaks for integral intensities (I_i_) and I_i_/I_600°C_ ratios of the diffraction peaks for the FePt (**a**,**b**) and CoPt (**c**,**d**) systems (in situ XRD).

Dependencies of the boundaries of the monophase region of platinum-rich FCC SS (Figure 8) on temperature were determined by a series of experiments, presented in [1]. Based on such experiments, dependencies between temperature and the integral intensities (I_i_) and I_i_/I_600°C_ ratios of the diffraction peaks for XRDPh of FCC of different compositions were carefully analyzed (here I_i_ and I_600°C_ are the integral intensity at the current temperature and at 600 °C, the temperature of formation of practically pure Pt phase). The results of the analysis are presented in Figure 9. Due to the fact that the LS value in the CoPt system was significantly higher than in the FePt system, the discussed effects in the former case were more significant.

Due to the inability to detect Fe-rich and Co-rich phases in nanoparticles using XRD, there were no defined positions of the temperature profiles of their compositions and, therefore, it was difficult to make quantitative conclusions based on the temperature dependencies of I_i_ and I_i_/I_600°C_ values. At the same time, the lever rule for the two-phase region can cause the values of both I_i_ and I_i_/I_600°C_ to decrease with increasing temperature when the systems are in equilibrium. When the temperature was below the phase transformation temperature, the dependence of I_i_ and I_i_/I_600°C_ values on temperature was very weak, since these dependencies depicted only the temperature dependence of the diffraction-scattering intensity [47]. In the series of experiments, when the Fe and Co contents in SS decreased, the temperature boundary of the region of sharp decrease of I_i_ and I_i_/I_600°C_ values should have shifted to the region of high temperatures, and the general effect of the decrease in these values would be less pronounced (Figure 8).

These expected effects were qualitatively observed in experiments (Figure 9), thus confirming that they were due to the formation of two-phase regions in FePt and CoPt systems. The kinetics of changes in Fe and Co contents when specimens heated gradually in the region of T ≤ 250 °C was revealed in the form of changes in the XRDPh compositions of FCC, which occurred for about 1 h after the temperature increase; as for CoPt, in accordance with the position of the temperature boundary of phase transformations (Figure 9), there were no discussed effects up to 200–250 °C; they were observed at higher temperatures, where the CLP value changed only due to thermal expansion. Due to the higher LS value of Co in CoPt, the effects themselves were far more pronounced in CoPt (as previously mentioned); the low LS value of Fe in FePt made it difficult to observe such dependencies in FePt systems.

As previously mentioned, a significant increase in CLP was observed when the temperature exceeded 390 °C after the induction period; that is, the platinum content increased in the solid solution. Such changes in the composition of XRDPh (FCC SS) could be attributed to the observed increase in crystallite sizes of the nanoparticles (Figure 5). The result of this was a slower phase transformation.

According to the tendency at cooling from 600 °C (Figure 9), the realization of equilibrium states was difficult (the process requires very long temperature control at low temperatures); however, the process was reversible, and at the defined times of temperature control, there was a tendency to return to the initial states of the systems.

Thus, based on the results described above and in [1], a model of low-temperature phase transformations in platinum-rich particles could be proposed as follows:

XRDPh (A1, Fe_a_Pt_1−a_) **→** XRDPh (A1, Fe*_m_*_×a−x_Pt*_m_*_−*m*×a+x_)+ XRNDPh-2 (Fe*_n_*_×a+y_Pt*_n_*_−*n*×a−y_), here *m* + *n* = 1, *m* ≤ 1, *n* ≤ 1. (12)

Precision analysis of the XRD patterns when the temperatures were below 390 °C also suggested the possibility of additional, difficult-to-register phase transformations in NPs; synchrotron radiation X-ray diffraction experiments are being planned to reveal their nature.

### 3.3. X-ray Non-Detectable Phases—XRNDPh-1 and XRNDPh-2

Since there were solubility limits of Fe and Co in FCC solid solution, it was reasonable to assume that when the Fe and Co contents in nanosystems were above the LS value, two phases were formed during the synthesis, i.e., XRDPh (FCC solid solution) and XRNDPh-1 (Fe- or Co-rich phase), and in another case when the XRNDPh-2 was formed through phase transformations by the heating of nanoalloys. Due to the nontriviality of such an assumption, it should be considered in more detail.

As shown in Figure 3, there were nanoparticles with sizes in the range of 2–4 nm (Figure 3l,m). The size distribution of such particles is shown in Figure 3p and corresponds to the size range estimated using SAXS (Figure 7c,d). A part of the nanoparticles has a banded structure, the feature of crystallites, with distances between lattice fringes of 2.4 Å, corresponding to the 110 direction in the 111 planes of the FCC lattice. Furthermore, their shapes are close to hexagons. This provides a basis for attributing these nanoparticles to XRDPh (FCC solid solution).

In addition to these NPs, there were spherical-like particles in the same size range and, at the limit of TEM resolution, shapeless small nanoparticles in the subnanoscale range without a banded structure (Figure 3l,m). The same size range in the specimens was also shown by the SAXS method (Figure 4c,d; the 1–2 nm range).

At the qualitative level, the presence of particularly low-sized particles was shown using sorbtometry. The measured values of the specific surface area of the specimens were 2 m^2^/g for Fe_7_Pt_93_ (i.e., C(Fe) < SL) and 34 m^2^/g for Fe_73_Pt_27_ (i.e., C(Fe) > SL).

There were at least two reasons why phases detected no XRD. The particles of the XRNDPhs were, in one sense, in the ultra-small size range that resulted in high broadening of diffraction peaks, which made registration difficult against the background of close-in-composition XRDPh peaks, or even practically amorphous. On the other hand, these particles had the lower efficiency of X-ray scattering, as XRNDPhs were platinum-depleted.

**Figure 10:**
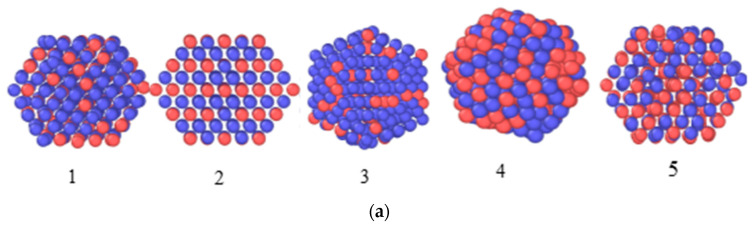

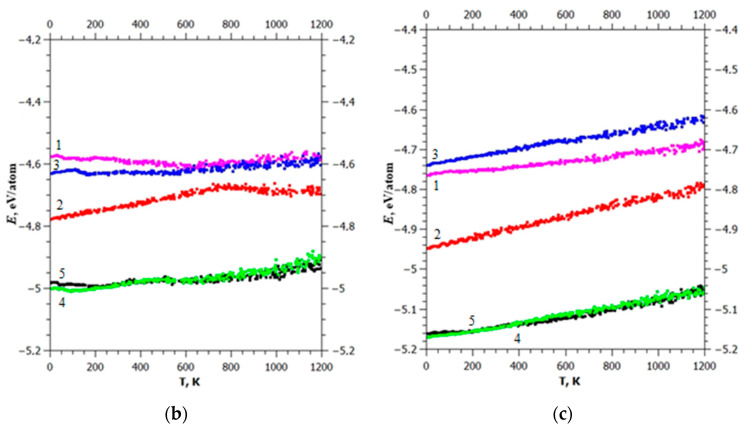
(**a**) Various habitus and structures of nanoclusters: 1—FePt_3_ cuboctahedron with A1-type structure, 2—FePt_3_ cuboctahedron with L1_2_-type structure, 3—FePt_3_ icosahedron with A1-type structure, 4—FePt icosahedron with A1-type structure, 5—FePt cuboctahedron with A1-type structure; dependence of total energy on temperature for clusters of 1.8 nm (309-atom) (**b**) and 2.8 nm (923-atom) (**c**).

The stability of FePt nanoclusters with A1- and L1_2_-type structures (Figure 1) and different habitus (Figure 10a) was calculated for nanoparticles with sizes 2–3 nm, similar to those registered by TEM and SAXS.

It can be seen (Figure 10b,c) that a closed-shell Fe-Pt cluster of 309 atoms (1.8 nm) and 923 atoms (2.80 nm) were characterized by similar patterns:

- A1 phase clusters (FCC SS) were the most stable, with no strong effect of the habitus on the stability;
- For the FePt_3_ composition, (ordered L1_2_) cubic shape was more stable.

Therefore, the results of the calculations showed that the cuboctahedral form was more stable at the synthesis temperatures of the samples, which agreed with the TEM data.

At the same time, according to the calculations, clusters with an ordered L1_2_ structure (especially small clusters of size below 2 nm) were also stable. It followed that when Fe and Co contents in nanosystems were above the LS value, XRNDPh-2 was most likely represented by particles in the size range of 2–4 nm with the L12 structure, as well as smaller clusters with the same structure and (or) amorphous arrays.

In the authors’ view, this follows from an earlier proposal discussed in [48], in terms of FePt_3_ formation at heating. As a result of in situ XRD and HR TEM studies, a complex interdependence between internal structure and heating mode for core-shell nanoparticles was shown. However, at the same time, no unconventional transformation at the heating of FePt_3(core)_ Pt_(shell)_ nanoparticles, observed in [48], were detected. Probably, this matter requires more research.

The observation of XRNDPh-2 particles was additionally complicated by the fact that, according to the HRTEM results, they were formed during phase transformation in the form of nanoparticles tightly adhering to the surface of NPs, or “absorbed” by the surface layers. Experiments are currently underway to characterize them by both XPS with Ar-etching of the specimen surface and synchrotron radiation diffractometry.

These results, as well as the results of the study of the features of phase transformations in the FePt and CoPt systems with higher Fe and Co contents by heating, will be presented in subsequent publications. 

## 4. Conclusions

This article’s research efforts sought to study the phase-structural properties (phase compositions, structure) and morphology of FePt and CoPt nanoparticles in the range of platinum-rich compositions obtained by reduction of precursor aqueous solutions with hydrazine hydrate. Those efforts were complemented by the calculation of the cluster’s stability via the molecular-dynamics method. 

The results ed the following:

- There is only an FCC-A1-type disordered platinum-rich solid solution (XRDPhs), according to the XRD method. The solubility limits of Fe and Co were determined (11.4 ± 0.7% and 17.5 ± 0.6%, respectively). The structural blockiness of the main fraction of crystals (4–10 nm) and the established distributions of their sizes were found. The presence of especially fine-dispersed particles of 1–4 nm was revealed, from which, probably, the main fractions were composed; Heterogeneity in the composition of crystals of the main fraction was found, i.e., there were inner (deep) regions enriched with platinum. Based on above results, the paper proposes a model of solid-solution formation during the reduction of components with significantly different redox potentials (Eh);
- There was formation along with the diffraction-registered phase and the X-ray non-detectable phase (XRNDPh-1) when the Fe and Co contents in the nanosystems were above the solubility limits, the presence of which were confirmed by the HRTEM and SAXS methods and which were composed, probably, of the crystallites (1–3 nm) and amorphous arrays;
- Heating of solid solutions with compositions C(Fe,Co) < LS above the threshold temperatures led to phase transformations, with the formation of Pt-rich XRDPh and XRNDPh-2 with high contents of Fe(Co). A model of phase transformation was proposed.

The results of the research presented in paper shed some light on the details of the phase transformations during heating of nanoalloys with higher Fe, Co contents, including equiatomic compositions. These processes lead to the formation of highly ordered FePt, CoPt intermetallic compounds with an L1_0_ structure of unique magnetic properties.

## Figures and Tables

**Table 1:** Chemical composition and lattice parameters of synthesized nanoparticles.

**System**	**FePt**					
C_Fe_^1^ as synthesized, mol.%	5	7	13	15	22	28
CLP, Å	3.907 ± 0.001	3.907 ± 0.001	3.9089 ± 0.0001	3.9032 ± 0.0004	3.897 ± 0.001	3.8985 ± 0.0004
C_Co_ ^2^ in FCC, mol. %	8.0	8.0	7.1	9.8	12.6	11.9
**System**	**CoPt**					
C_Co_^1^ as synthesized, mol.%	6	11	14	20	26	31
CLP, Å	3.902 ± 0.002	3.898 ± 0.001	3.898 ± 0.001	3.895 ± 0.001	3.876 ± 0.001	3.870 ± 0.002
C_Co_^2^ in FCC, mol. %	7.3	8.6	8.9	10.2	15.9	17.9

^1^ According to ICP-OES. ^2^ According to XRD.

## Data Availability

The data presented in this study are available on request from the corresponding author.

## Supplementary Materials

The following supporting information can be downloaded at: https://www.mdpi.com/article/10.3390/ma16237312/s1, Figure S1: XRD patterns of Fe5Pt95 (red) and Co14Pt86 (black) when specimens were heated for 2 h at 390 °C; Figure S2: The transformation of (311) peak during heating (red lines) and cooling (blue lines) (Fe5Pt95 (a) and Co14Pt86 (b)).

## References

[1] Zakharov N.S., Tikchonova I.N., Zakharov Y.A., Popova A.N., Pugachev V.M., Russakov D.M. (2022). Study of the Pt-rich nanostructured FePt and CoPt alloys: Oddities of phase composition. Lett. Mater..

[2] Zakharov N.S., Pugachev V.M., Popova A.N. (2021). Platinum-rich solid solution in nanostructured FePt system. Chem. Sustain. Dev..

[3] Pugachev V.M., Zakharov Y.A., Popova A.N., Russakov D.M., Zakharov N.S. (2021). Phase transformations of the nanostructured iron-platinum system upon heating. J. Phys. Conf. Ser..

[4] Pei W., Zhao D., Wu C., Sun Z., Liu C., Wang X., Zheng J., Yan M., Wang J., Wang Q. (2020). Direct Synthesis of L 10-FePt Nanoparticles with High Coercivity via Pb Addition for Applications in Permanent Magnets and Catalysts. ACS Appl. Nano Mater..

[5] Poudyal N., Chaubey G.S., Rong C.B., Liu J.P. (2009). Shape control of FePt nanocrystals. J. Appl. Phys..

[6] Wittig J.E., Bentley J., Allard L.F. (2017). In situ investigation of ordering phase transformations in FePt magnetic nanoparticles. Ultramicroscopy.

[7] Massalski T.B. (1992). Binary Alloys Phase Diagram.

[8] Okamoto H. (1993). Phase Diagrams of Binary Iron Alloys.

[9] Schneider S., Pohl D., Löffler S., Rusz J., Kasinathan D., Schattschneider P., Schultz L., Rellinghaus B. (2016). Magnetic properties of single nanomagnets: Electron energy-loss magnetic chiral dichroism on FePt nanoparticles. Ultramicroscopy.

[10] Zhou C., Schulthess T.C., Mryasov O.N. (2007). Magnetic anisotropy of FePt nanoparticles: Temperature-dependent free energy barrier for switching. IEEE Trans. Magn..

[11] Lyberatos A., Weller D., Parker G.J., Stipe B.C. (2012). Size dependence of the Curie temperature of L10-FePt nanoparticles. J. Appl. Phys..

[12] Luo H.B., Xia W.X., Ruban A.V., Du J., Zhang J., Liu J.P., Yan A. (2014). Effect of stoichiometry on the magnetocrystalline anisotropy of Fe–Pt and Co–Pt from first-principles calculation. J. Condens. Matter Phys..

[13] Natekar N.A., Hsu W.H., Victora R.H. (2017). Calculated dependence of FePt damping on external field magnitude and direction. AIP Adv..

[14] Chen S., Andre P. (2012). Colloidal syntheses of FePt nanoparticles. Int. J. Nanotechnol..

[15] Li J., Sun S. (2019). Intermetallic nanoparticles: Synthetic control and their enhanced electrocatalysis. Acc. Chem. Res..

[16] Chrobak A. (2022). High and ultra-high coercive materials in spring-exchange systems—Review, simulations and perspective. Materials.

[17] Dalavi S.B., Panda R.N. (2017). Observation of high coercive fields in chemically synthesized coated Fe-Pt nanostructures. J. Magn. Magn. Mater..

[18] Gutfleisch O., Lyubina J., Müller K.H., Schultz L. (2005). FePt hard magnets. Adv. Eng. Mater..

[19] Rellinghaus B., Stappert S., Acet M., Wassermann E.F. (2003). Magnetic properties of FePt nanoparticles. J. Magn. Magn. Mater..

[20] Chou S.W., Zhu C.L., Neeleshwar S., Chen C.L., Chen Y.Y., Chen C.C. (2009). Controlled growth and magnetic property of FePt nanostructure: Cuboctahedron, octapod, truncated cube, and cube. Chem. Mater..

[21] Komorida Y., Mito M., Deguchi H., Takagi S., Iwamoto T., Kitamoto Y. (2010). Pressure-induced enhancement of the blocking temperature in FePt nanoparticles. J. Phys. Conf. Ser..

[22] Liang J., Ma F., Hwang S., Wang X., Sokolowski J., Li Q., Wu G., Su D. (2019). Atomic Arrangement Engineering of Metallic Nanocrystals for Energy-Conversion Electrocatalysis. Joule.

[23] Li Q., Wu L., Wu G., Su D., Lv H., Zhang S., Zhu W., Casimir A., Zhu H., Mendoza-Garcia A. (2015). New Approach to Fully Ordered fct-FePt Nanoparticles for Much Enhanced Electrocatalysis in Acid. Nano Lett..

[24] Wen Z., Wang Y., Wang C., Jiang M., Li H., Ren Y., Qin G. (2022). Redetermination of the Fe–Pt phase diagram by using diffusion couple technique combined with key alloys. Int. J. Mater. Res..

[25] Yang B., Asta M., Mryasov O.N., Klemmer T.J., Chantrell R.W. (2005). Equilibrium Monte Carlo simulations of A1–L10 ordering in FePt nanoparticles. Scr. Mater..

[26] Zakharov N.S., Popova A.N., Zakharov Y.A., Pugachev V.M., Russakov D.M. (2022). Transmission Electron Microscopy: Study of the Bimetallic Nanoparticle Features. Russ. J. Phys. Chem..

[27] Yakubik D.G., Sadykova L.R., Zakharov Y.A., Zakharov N.S., Popova A.N., Pugachev V.M. (2022). Stability of FePt, FePt3 Nanoclusters of Different Habits. Eurasian Chem.-Technol. J..

[28] Zakharov Y.A., Pugachev V.M., Korchuganova K.A., Ponomarchuk Y.V., Larichev T.A. (2020). Analysis of phase composition and CSR sizes in non-equilibrium nanostructured systems Fe-Co and Ni-Cu using diffraction maxima simulations in a doublet radiation. J. Struc. Chem..

[29] Whyte T.E., Kirklin P.W., Gould R.W., Heinemann H. (1972). Small angle X-ray scattering investigation of platinum metal dispersions on alumina catalysts. J. Catal..

[30] White H.W. (1990). Particle size distribution that cannot be distinguished by their integral moments. J. Colloid Interface Sci..

[31] Li T., Senesi A.J., Lee B. (2016). Small Angle X-ray Scattering for Nanoparticle Research. Chem. Rev..

[32] Dodonov V.G., Zakharov Y.A., Pugachev V.M., Vasiljeva O.V. (2016). Determination of the surface structure peculiarities of nanoscale metal particles via small-angle X-ray scattering. Inorg. Mater. Appl. Res..

[33] Ivanova N., Lobanov A., Andyyakova A., Zakharov Y., Popova A., Kolmykov R. (2020). The electrochemical synthesis and investigation of nanostructured Fe-Pt and Co-Pt systems. IOP Conf. Ser. Mater. Sci. Eng..

[34] LAMMPS. www.lammps.org.

[35] Lee B., Baskes M.I., Kim H., Cho Y.K. (2001). Second nearest-neighbor modified embedded atom method potentials for bcc transition metals. Phys. Rev. B.

[36] Interatomic Potentials Repository. https://www.ctcms.nist.gov/potentials/.

[37] Liu X., Wang H., Zuo S., Zhang T., Dong Y., Li D., Jiang C. (2020). Dispersible and manipulable magnetic L10-FePt nanoparticles. Nanoscale.

[38] Dmitrieva O., Rellinghaus B., Kästner J., Dumpich G. (2007). Quantitative structure analysis of L10-ordered FePt nanoparticles by HRTEM. J. Cryst. Growth.

[39] Dai Z.R., Sun S., Wang Z.L. (2001). Phase transformation, coalescence, and twinning of monodisperse FePt nanocrystals. Nano Lett..

[40] Bard A.J., Parsons R., Jordan J. (1985). Standard Potentials in Aqueous Solutions.

[41] Sahyun M.R.V. (1978). Towards a quantum chemical model of the photographic process. Photogr. Sci. Engng..

[42] Belous V.M. (1997). Review of luminescence studies on latent image formation in silver halide emulsions. J. Imaging Sci. Technol..

[43] Hamilton J.F. (1988). The silver halide photographic process. Adv. Phys..

[44] Yang J., Zhou W., Cheng C.H., Lee J.Y., Liu Z. (2010). Pt-decorated PdFe nanoparticles as methanol-tolerant oxygen reduction electrocatalyst. ACS Appl. Mater. Interfaces.

[45] Cotton S. (1997). Chemistry of Precious Metals.

[46] IUPAC-NIST Solubility Database (Version 1.1), NIST Standard Reference Database 106. 2012. Office of Data and Informatics of the National Institute of Standards and Technology (NIST), USA. https://srdata.nist.gov/solubility.

[47] Koch E. (2006). International Tables for Crystallography.

[48] Chen X., Zhang S., Li C., Liu Z., Sun X., Cheng S., Zakharov D.N., Hwang S., Zhu Y., Fang J. (2022). Composition-dependent ordering transformations in Pt–Fe nanoalloys. Proc. Natl. Acad. Sci. USA.

