# Supplementary material for: Morphology and Phase Compositions of FePt and CoPt Nanoparticles Enriched with Noble Metal
Source: Materials (Basel). 2023 Nov 24;16(23):7312. doi: 10.3390/ma16237312 (PMC10707323; doi:10.3390/ma16237312)
Supplement: Supplementary file 1 [file materials-16-07312-s001.zip › materials-2701646-supplementary.pdf]

Supplementary

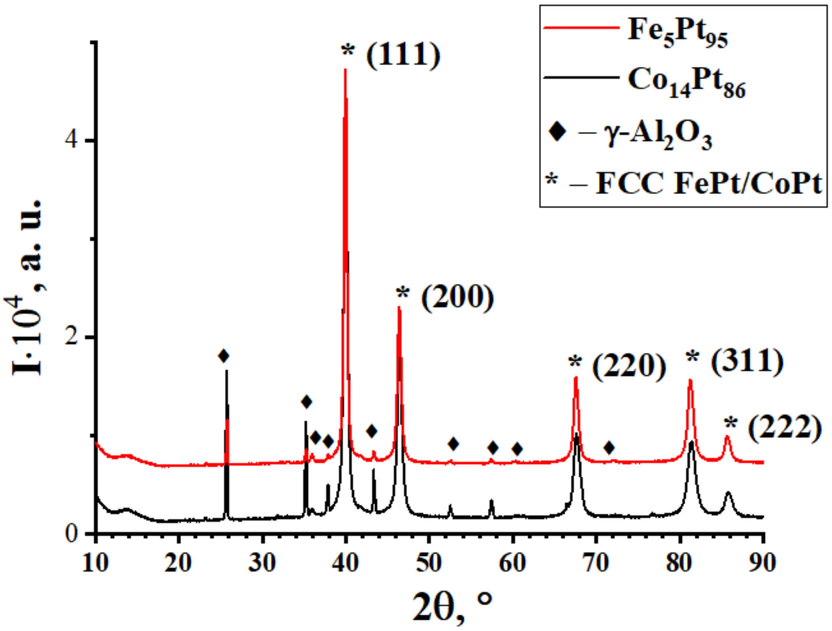

S1. XRD patterns of Fe_5Pt_{95} (red) and $Co_{14}Pt_{86}$ (black) when specimens were heated for 2 hours at 390 °C.

◆ – sample-holder, * – fcc

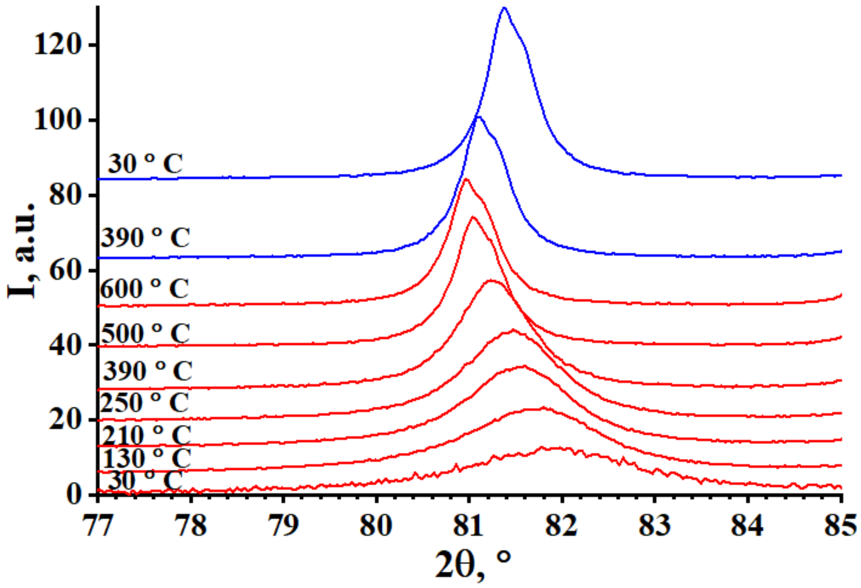

(a)

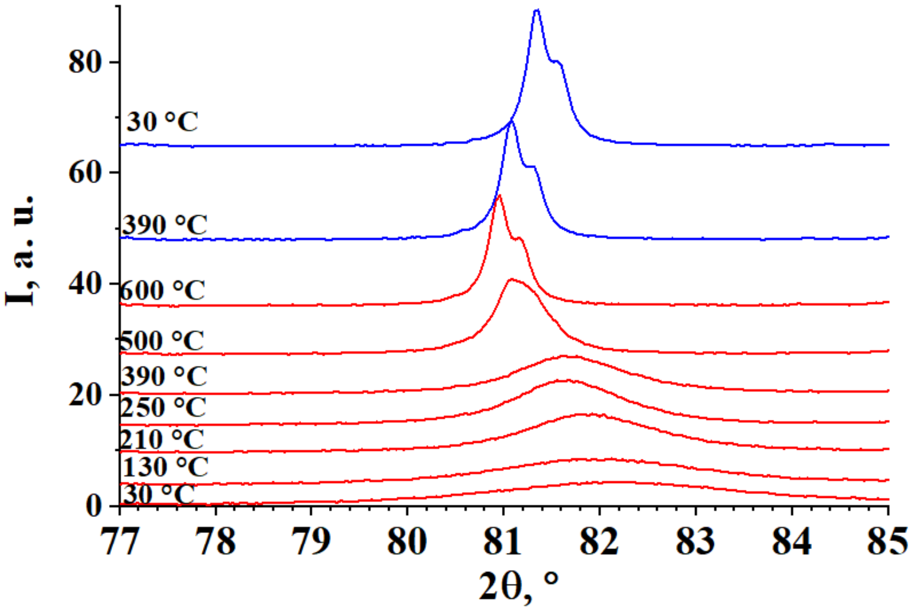

(b)

S2. The transformation of (311) peak during heating (red lines) and cooling (blue lines) (Fe_5Pt_{95} **(a)** and $Co_{14}Pt_{86}$ **(b)**).
